# Supplementary material for: Reference Excitation Energies of Increasingly Large Molecules: A QMC Study of Cyanine Dyes
Source: J Chem Theory Comput. 2022 Jan 26;18(2):1089–95. doi: 10.1021/acs.jctc.1c01162 (PMC8830039; doi:10.1021/acs.jctc.1c01162)
Supplement: Supplementary file 1 — ct1c01162_si_001.pdf [file ct1c01162_si_001.pdf]

Supporting Information: Reference quantum Monte Carlo calculations for the excited states of cyanine dyes

Alice Cuzzocrea,[†] Saverio Moroni,[*,‡] Anthony Scemama,[*,¶] and Claudia Filippi[*,†]

†*MESA+ Institute for Nanotechnology, University of Twente, P.O. Box 217, 7500 AE Enschede, The Netherlands*

‡*CNR-IOM DEMOCRITOS, Istituto Officina dei Materiali, and SISSA Scuola Internazionale Superiore di Studi Avanzati, Via Bonomea 265, I-34136 Trieste, Italy*

¶*Laboratoire de Chimie et Physique Quantiques, Université de Toulouse, CNRS, UPS, France*

E-mail: moroni@democritos.it; scemama@irsamc.ups-tlse.fr; c.filippi@utwente.nl

S1 Dependence of excitation energies on basis set and pseudopotentials

Using CC3 calculations, we explore here the errors arising from the use of pseudopotentials and the choice of basis set. As shown in Table S1 for CN3, CN5, and CN7, the impact of the pseudopotential on the CC3 excitation energy is very small (about 0.02 eV) and, while the use of the maug-cc-pVDZ basis is not sufficient for CN3, this basis set is appropriate for the larger cyanine molecules, starting from CN5.

Table S1: CC3 excitation energies (eV) computed with the BFD pseudopotentials and in an all-electron calculation with different basis sets.

CN3		
Basis	BFD	All-electron
maug-cc-pVDZ	7.268	–
aug-cc-pVDZ	7.182	7.202
aug-cc-pVTZ	–	7.190
CN5		
maug-cc-pVDZ	4.828	–
aug-cc-pVDZ	4.834	4.851
aug-cc-pVTZ	–	4.844
CN7		
maug-cc-pVDZ	3.635	–
aug-cc-pVDZ	3.654	3.669
CN9		
maug-cc-pVDZ	2.932	–
aug-cc-pVDZ	2.956	2.970

S2 CIPSI and QMC results for CN3

In Table S2, we report the CI energies, PT2 energies, and CI variances for the CIPSI expansions of CN3 used in the QMC-CIPSI calculations of Table III of the main text. The values are relative to single-state CIPSI wave functions of increasing size, that are separately expanded for the ground and excited states until when the corresponding CI variances match. We consider the CI variances matched if their difference is less than 0.002 a.u.

In Figure S1, we plot the CI excitation energies of CN3 versus the total (GS+ES) number of determinants. The excitation energies are obtained by matching the variance or the PT2 energy correction. Even though the PT2-matched excitation energy converges faster for small expansions, matching the CI variance automatically leads to expansions with also matched PT2 energy correction beyond few thousands total (GS+ES) determinants. We also show that the use of CAS(4,3) or CAS(4,6) optimized orbitals does not have an impact on the CIPSI convergence.

Going to larger CIPSI expansions, we can also compute the extrapolated FCI excitation energy by fitting the ground- and excited-state CI energies versus the renormalized PT2 (rPT2) energy

Table S2: CI energies, CI variances, and PT2 energy corrections (a.u.) of the ground- and excited-state CIPSI expansions of CN3. The expansions are matched according to the iso-variance criterion. The CI excitation energy ΔE_{CI} (eV) is also listed. We use the orbitals obtained from a CASSCF(6,10) calculation of symmetry A_1 and B_1 for the ground (GS) and excited (ES) states, respectively. The BFD pseudopotentials and corresponding basis sets are used.

No. det		E_{CI}		ΔE_{CI} (eV)	PT2		ΔPT2	σ^2_{CI}		$\Delta\sigma^2_{CI}$
GS	ES	GS	ES		GS	ES		GS	ES	
maug-cc-pVDZ basis set										
220	300	-27.7557	-27.4770	7.584	-0.4625	-0.4705	-0.0080	1.5994	1.5984	0.0010
602	1254	-27.7898	-27.5141	7.501	-0.4167	-0.4217	-0.0050	1.5050	1.5039	0.0011
1028	2446	-27.8124	-27.5396	7.422	-0.3879	-0.3905	-0.0026	1.4391	1.4373	0.0018
2511	7092	-27.8618	-27.5907	7.376	-0.3296	-0.3300	-0.0004	1.2905	1.2885	0.0020
5005	14696	-27.9085	-27.6363	7.408	-0.2773	-0.2799	-0.0026	1.1420	1.1395	0.0025
6508	20144	-27.9285	-27.6578	7.366	-0.2557	-0.2575	-0.0018	1.0733	1.0744	-0.0011
aug-cc-pVTZ basis set										
1483	3246	-27.8102	-27.5367	7.440	-0.5197	-0.5238	-0.0041	2.5206	2.5195	0.0011
2569	6432	-27.8291	-27.5574	7.395	-0.4945	-0.4976	-0.0031	2.4594	2.4587	0.0007
4451	12008	-27.8510	-27.5800	7.373	-0.4672	-0.4698	-0.0027	2.3868	2.3881	0.0013

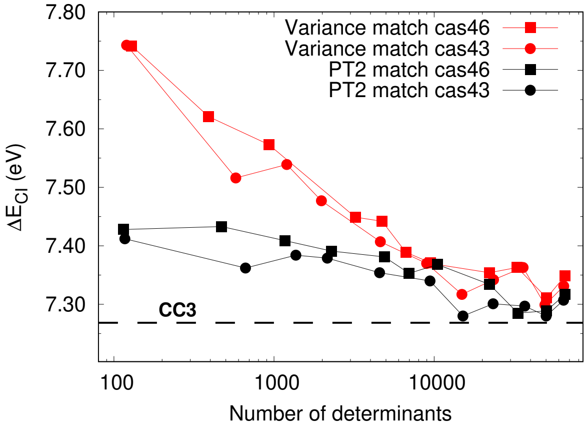

Figure S1: CI excitation energies of CN3 computed for expansions of increasing size with matched variance or PT2 energy correction and two different CASSCF orbital sets generated with a CAS(4,3) and a CAS(4,6) calculation. The BFD pseudopotentials and the corresponding maug-cc-pVDZ basis set is used for all calculations.

corrections,[1] which become zero in the FCI limit. In Fig. S2, we show the convergence of the CI energies as well as their polynomial fits, which are performed over the interval [-0.1:0] of rPT2 values. We chose to extrapolate using the rPT2 energy corrections (instead of the PT2 ones) since the behavior is more linear as also shown in the Figure. The estimated FCI/maug-cc-pVDZ energy is 7.25 eV, close to the CC3/maug-cc-pVDZ (BFD) value of 7.27 eV.

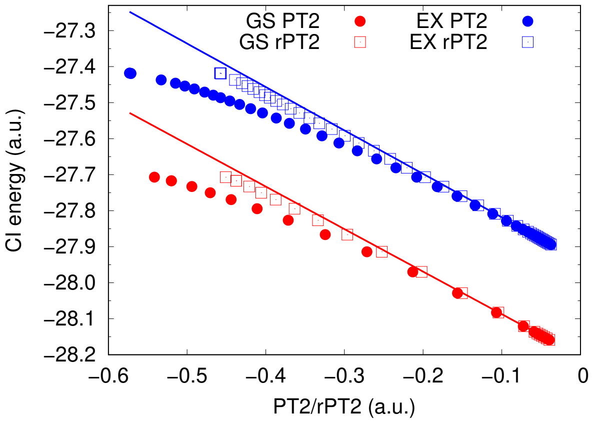

Figure S2: Ground- and excited-state CI energies of CN3 versus the rPT2 energy corrections and corresponding fits. The CI energies are also plotted against the PT2 energy corrections.

In Table S3, we list the VMC and DMC excitation energies of CN3 computed with different determinantal components in the wave functions and with two basis sets. We employ CIPSI expansions with matched CI variances.

Table S3: VMC and DMC total energies (a.u.) and excitation energies (eV) of CN3 obtained for different wave functions. All the variational parameters (Jastrow, orbital, and CI coefficients) are optimized in energy minimization. We employ the BFD maug-cc-pVDZ basis set, unless we label the calculation with "Ta" which denotes the use of the BFD aug-cc-pVTZ basis set.

WF	No. det		No. parm		E_{VMC}		ΔE_{VMC}	E_{DMC}		ΔE_{DMC}
	GS	ES	GS	ES	GS	ES		GS	ES	
HF/HL	1	2	220	230	-28.3005(3)	-28.0238(3)	7.529(13)	-28.3666(2)	-28.0934(2)	7.435(8)
CAS(4,3)	5	4	232	230	-28.3057(2)	-28.0238(3)	7.667(11)	-28.3693(2)	-28.0941(2)	7.488(8)
CAS(4,6)	113	112	302	293	-28.3077(4)	-28.0302(3)	7.552(13)	-28.3694(2)	-28.0959(2)	7.442(7)
CAS(4,6)-Ta	113	112	1008	999	-28.3165(3)	-28.0412(3)	7.492(13)	-28.3706(2)	-28.0985(2)	7.406(7)
CIPSI	220	300	690	880	-28.3201(2)	-28.0483(2)	7.396(08)	-28.3715(2)	-28.1039(2)	7.283(8)
	602	1254	1179	1893	-28.3334(3)	-28.0625(3)	7.372(11)	-28.3752(2)	-28.1074(2)	7.288(7)
	1028	2446	1653	2293	-28.3375(3)	-28.0682(3)	7.326(10)	-28.3768(2)	-28.1095(2)	7.274(7)
	2511	7092	2624	3872	-28.3444(3)	-28.0776(3)	7.262(10)	-28.3806(2)	-28.1140(2)	7.254(7)
	5005	14696	3688	6145	-28.3502(3)	-28.0835(3)	7.257(10)	-28.3830(2)	-28.1168(2)	7.242(8)
	6508	20144	4234	7648	-28.3526(2)	-28.0861(2)	7.252(07)	-28.3843(2)	-28.1180(2)	7.245(6)
CIPSI-Ta	1483	3246	8395	10445	-28.3427(2)	-28.0760(3)	7.257(09)	-28.3773(2)	-28.1114(2)	7.236(7)
	2569	6432	10988	14765	-28.3461(2)	-28.0803(2)	7.232(09)	-28.3783(2)	-28.1128(2)	7.224(7)
	4451	12008	14780	19237	-28.3515(2)	-28.0859(2)	7.228(09)	-28.3802(2)	-28.1146(2)	7.227(6)
CC3/aug-cc-pVDZ										7.20
CC3/aug-cc-pVTZ										7.19

In Fig. S3, we plot the VMC/maug-cc-pVDZ energies of the two states versus their VMC variances. As proposed in Refs. 2,3, we fit the VMC energies for the ground and excited state against the corresponding VMC variances and estimate the excitation energy as the difference

$\Delta E^{\text{fit}}_{\text{VMC}} = E^{\text{fit}}_{\text{ES}}(\sigma^2) - E^{\text{fit}}_{\text{GS}}(\sigma^2)$ of the fits of the energies. The resulting excitation energy is consistently overestimated and, as the variance becomes smaller, further departs from the reference CC3 value.

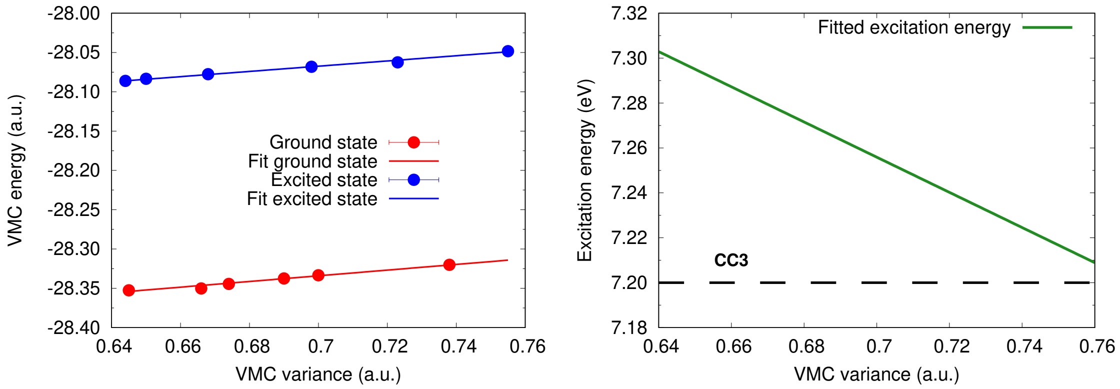

Figure S3: VMC ground- and excited-state energies versus the corresponding VMC variances (left) and resulting fitted excitation energy (right). The reference CC3/aug-cc-pVDZ value is shown.

S3 CIPSI results for all molecules

In Fig. S4, we show the convergence as a function of the total number of determinants of the CI excitation energies of CN3–CN15 obtained with ground- and excited-state CIPSI expansions with either matched PT2 energy corrections or CI variances. The iso-variance procedure leads to a faster convergence of the CI excitation energy for the cyanine molecules larger than CN3 and is chosen to generate the CIPSI expansions for the calculations of the QMC excitation energies of all molecules. For all the cases in Fig. S4, the CIPSI expansions are performed on CASSCF optimized orbitals.

For CN17 and CN19, we perform instead the CIPSI expansions on HF orbitals and show the CI convergence of their excitation energies for the iso-variance procedure[1] in Fig. S5 and further discuss the use of HF orbitals in Section S3.1.

In Table S4, we list the CI energies, CI variances, and PT2 energy corrections of the CIPSI expansions used in the QMC calculations for CN5–CN19 (the data for CN3 are reported above in Tab. S2).

[1]We do not show the iso-PT2 curves since, in the shown determinantal range, the iso-PT2 criterion leads to excitation energies very far from convergence.

Table S4: CI energies, CI variances, and PT2 energy corrections (a.u.) of the ground- and excited-state CIPSI expansions. The CI excitation energy (ΔE_{CI}) is given in eV. The basis set is the maug-cc-pVDZ.

System	No. det		E_{CI}		ΔE_{CI} (eV)	δE_{PT2}		$\Delta\delta E_{PT2}$	σ^2_{CI}		$\Delta\sigma^2_{CI}$
	GS	ES	GS	ES		GS	ES		GS	ES	
					PT2 matched						
**CN7**	2147	6038	-52.2231	-52.0819	3.841	-0.9157	-0.9152	-0.0005	3.1305	3.1449	-0.0144
					Variance matched						
**CN5**[a]	376	1094	-39.9732	-39.7922	4.925	-0.7227	-0.7200	0.0027	2.4041	2.4063	0.0022
	1344	4382	-40.0187	-39.8379	4.920	-0.6568	-0.6563	0.0005	2.2921	2.2964	0.0043
	2460	8782	-40.0480	-39.8701	4.841	-0.6170	-0.6145	0.0025	2.2164	2.2157	-0.0007
	3913	14114	-40.0750	-39.8975	4.830	-0.5826	-0.5788	0.0038	2.1427	2.1415	-0.0012
**CN7**	1871	6038	-52.2170	-52.0819	3.676	-0.9249	-0.9152	-0.0097	3.1453	3.1449	0.0005
	5578	18300	-52.2715	-52.1364	3.677	-0.8469	-0.8389	-0.0080	3.0125	3.0108	0.0017
**CN9**	1289	3372	-64.3840	-64.2724	3.035	-1.2322	-1.2156	-0.0166	4.0669	4.0684	0.0016
	2120	6360	-64.4021	-64.2928	2.975	-1.2009	-1.1834	-0.0175	4.0228	4.0226	0.0002
**CN11**	1071	2970	-76.5714	-76.4777	2.550	-1.5034	-1.4791	0.0244	4.9233	4.9242	-0.0009
	1812	5634	-76.5868	-76.4956	2.480	-1.4748	-1.4486	0.0261	4.8868	4.8849	0.0020
	2284	6896	-76.5950	-76.5026	2.515	-1.4599	-1.4373	0.0226	4.8675	4.8680	-0.0005
**CN13**	714	1810	-88.7502	-88.6676	2.247	-1.7897	-1.7607	0.0290	5.7972	5.7983	0.0011
	1406	4464	-88.7687	-88.6907	2.122	-1.7491	-1.7155	0.0336	5.7533	5.7514	-0.0019
**CN15**	1356	4434	-100.9602	-100.8909	1.886	-2.0056	-1.9686	0.0370	6.5999	6.5985	-0.0014
	2241	7996	-100.9766	-100.9074	1.883	-1.9701	-1.9357	0.0344	6.5582	6.5590	0.0008
**CN17**	559	2028	-113.0822	-113.0171	1.773	-2.4096	-2.3597	0.0499	7.5429	7.5409	0.0020
	943	3288	-113.0944	-113.0291	1.777	-2.3791	-2.3322	0.0469	7.5213	7.5209	0.0004
	1384	5068	-113.1043	-113.0411	1.720	-2.3560	-2.3056	0.0504	7.5013	7.5007	-0.0007
**CN19**	515	1886	-125.2638	-125.2039	1.630	-2.6943	-2.6371	0.0572	8.3908	8.3921	0.0013
	1487	4806	-125.2876	-125.2265	1.662	-2.6299	-2.5798	0.0501	8.3467	8.3491	0.0024

[a] Data from our work in Ref. 4

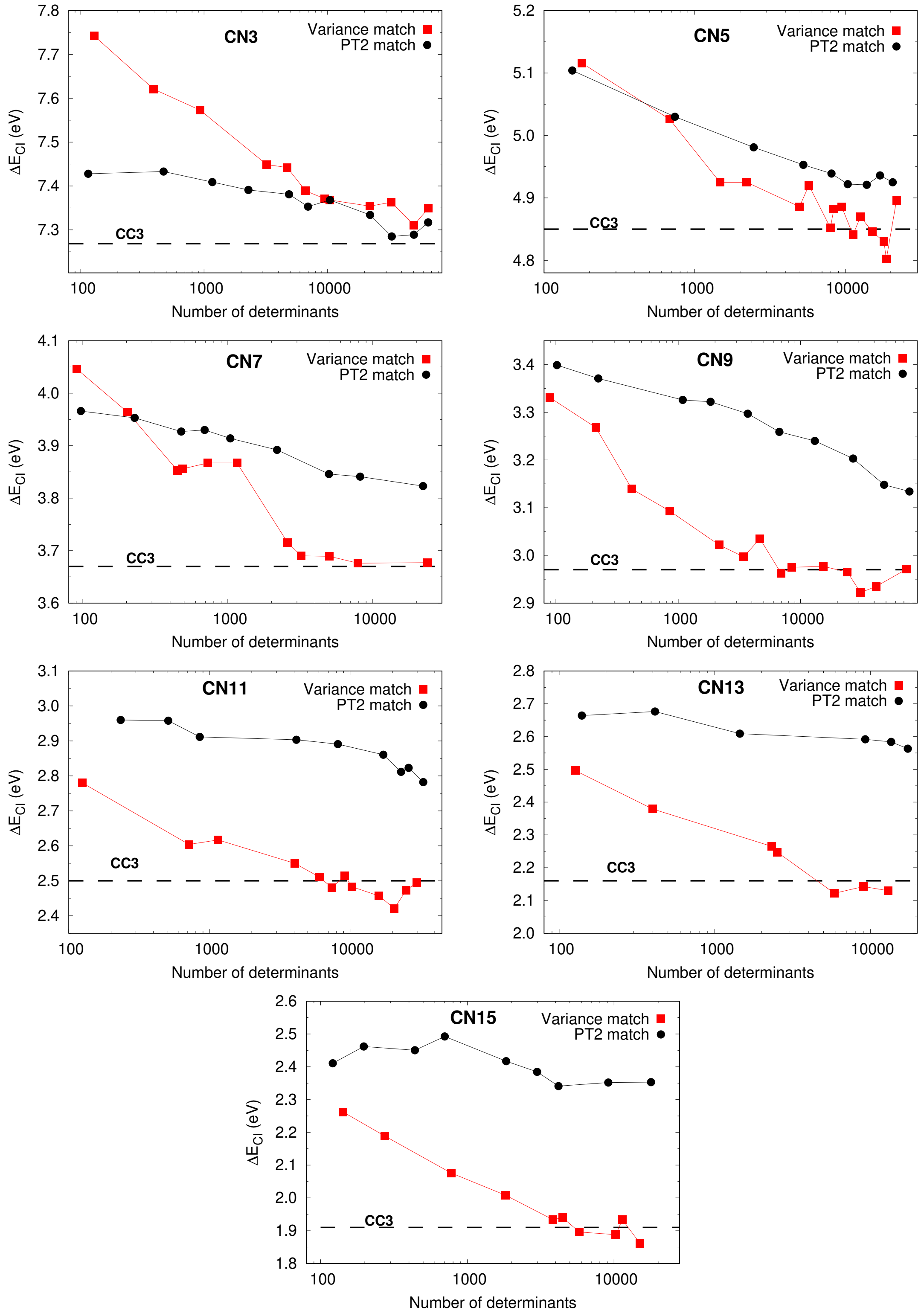

Figure S4: CI excitation energies of CN3–CN15 for iso-PT2 or iso-variance CIPSI expansions versus the total (GS+ES) number of determinants.

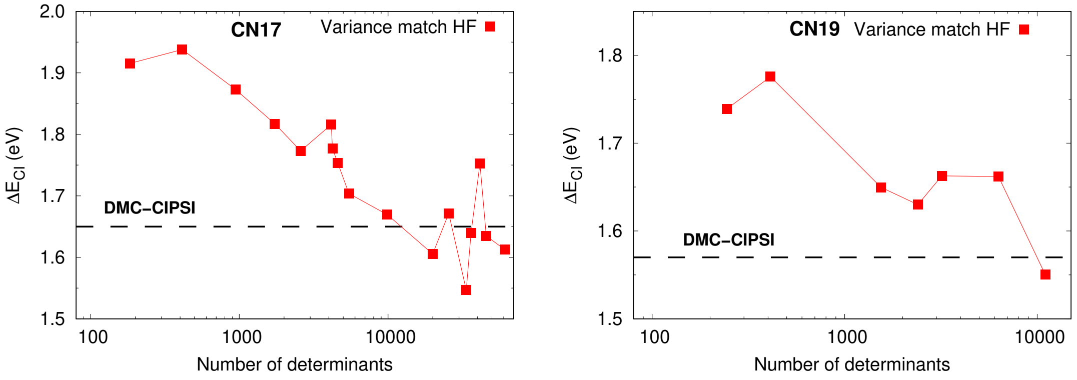

Figure S5: CI excitation energies of CN17 and CN19 for iso-variance CIPSI expansions performed on HF orbitals, versus the total (GS+ES) number of determinants.

S3.1 Use of HF orbitals in CIPSI expansions

Since, for large molecules, the CASSCF computation becomes quite expensive, we have used HF orbitals to perform the CIPSI calculation for CN17 and CN19. Here, we validate this choice with two different tests.

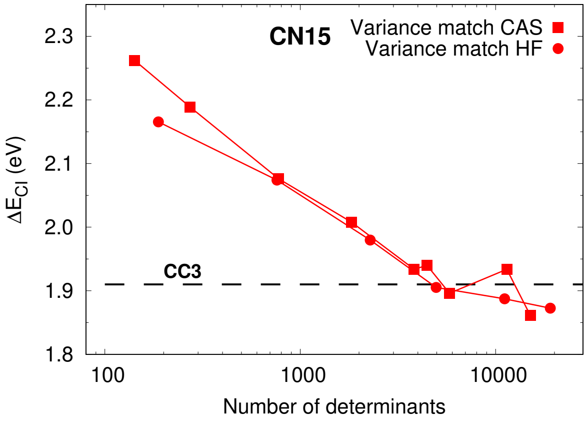

Figure S6: Convergence of variance-matched CI excitation energies of CN15 obtained with CAS(16,15) and HF orbitals, versus the total (GS+ES) number of determinants.

In Fig. S6, we compare the convergence of the iso-variance CI excitation energy of CN15 obtained with expansions on CASSCF and HF orbitals. We observe a very similar convergence for the two different orbitals, a finding which makes us confident in the use of HF orbitals for the larger chains. As an additional test, in Table S5, we report the QMC excitation energies of CN3, obtained with CIPSI determinantal expansions on CASSCF or HF orbitals. We find that, both at the VMC and the DMC level, the results obtained with expansions on HF orbitals are compatible with the CASSCF-based results with a similar number of determinants.

Table S5: VMC and DMC total energies (a.u.) and excitation energies (eV) of CN3 obtained with CIPSI expansions on either CASSCF or HF orbitals. All the variational parameters (Jastrow, orbital, and CI coefficients) are optimized in energy minimization. The maug-cc-pVDZ basis set is employed.

Starting orb.	No. det		No. parm		E_{VMC}		ΔE_{VMC}	E_{DMC}		ΔE_{DMC}
CASSCF	2511	7092	2624	3872	-28.3444(3)	-28.0776(3)	7.262(10)	-28.3806(2)	-28.1140(2)	7.254(7)
HF	2525	7374	2587	4066	-28.3452(3)	-28.0775(3)	7.282(10)	-28.3808(2)	-28.1140(1)	7.259(6)

S4 QMC results

In Table S6, we present the VMC and DMC total and vertical excitation energies obtained with the CIPSI expansions. In addition to the "best" excitation energies presented in the main text, we report more estimates obtained with different wave functions. For CN7, we perform QMC calculations both for CIPSI expansions with matched PT2 corrections and variances, while we only employ the iso-variance wave functions for the other molecules.

For CN7, for a similar number of determinants, the iso-PT2 selection gives slightly less accurate VMC excitation energies than the iso-variance case. However, at the DMC level, the differences are cured and the excitation energies are compatible.

Table S6: VMC and DMC total energies (a.u.) and excitation energies (eV) of cyanine dyes obtained for CIPSI wave functions. All the variational parameters (Jastrow, orbital, and CI coefficients) are optimized in energy minimization. The maug-cc-pVDZ basis set is employed.

System	No. det		No. parm		E_{VMC}		ΔE_{VMC}	E_{DMC}		ΔE_{DMC}
	GS	ES	GS	ES	GS	ES		GS	ES	
PT2 matched										
**CN7**	2147	6038	4349	6613	-53.4187(4)	-53.2810(3)	3.748(14)	-53.5172(2)	-53.3807(2)	3.714(8)
Variance matched										
**CN5**[a]	376	1094	1567	2609	-40.8646(3)	-40.6842(3)	4.908(12)	-40.9467(3)	-40.7665(3)	4.905(10)
	1344	4382	2478	4531	-40.8798(3)	-40.7013(3)	4.857(13)	-40.9502(2)	-40.7711(2)	4.872(09)
	2460	8782	3555	6561	-40.8896(3)	-40.7099(3)	4.890(12)	-40.9532(2)	-40.7748(2)	4.856(09)
	3913	14114	4842	8312	-40.8941(2)	-40.7167(3)	4.828(11)	-40.9559(2)	-40.7775(2)	4.856(08)
**CN7**	1871	6038	4105	6613	-53.4162(4)	-53.2810(3)	3.681(15)	-53.5170(2)	-53.3807(2)	3.708(8)
	5578	18300	7783	12098	-53.4338(3)	-53.2996(3)	3.651(12)	-53.5227(2)	-53.3883(2)	3.656(8)
**CN9**	132	286	2074	2268	-65.9167(4)	-65.8031(4)	3.090(15)	-66.0727(3)	-65.9618(3)	3.019(11)
	1289	3372	4785	7272	-65.9374(3)	-65.8255(3)	3.043(12)	-66.0772(3)	-65.9674(3)	2.989(10)
	2120	6360	5741	9345	-65.9482(5)	-65.8373(3)	3.031(12)	-66.0800(3)	-65.9704(3)	2.984(10)
**CN11**	1071	2970	5320	7403	-78.4706(3)	-78.3726(4)	2.668(13)	-78.6406(3)	-78.5471(3)	2.544(11)
	1812	5634	6606	10453	-78.4749(4)	-78.3795(3)	2.598(13)	-78.6422(3)	-78.5479(2)	2.566(10)
	2284	6896	7412	11729	-78.4777(4)	-78.3841(4)	2.547(14)	-78.6441(3)	-78.5507(3)	2.542(11)
**CN13**	714	1810	5306	5985	-90.9883(4)	-90.9056(4)	2.250(14)	-91.1986(3)	-91.1206(3)	2.124(11)
	1406	4464	7351	10297	-90.9966(4)	-90.9165(4)	2.180(14)	-91.2013(3)	-91.1224(3)	2.147(10)
**CN15**	1356	4434	7549	11347	-103.5244(3)	-103.4538(3)	1.920(12)	-103.7647(3)	-103.6934(3)	1.938(11)
	2241	7996	9951	15479	-103.5349(3)	-103.4668(3)	1.854(12)	-103.7674(3)	-103.6975(3)	1.902(11)
**CN17**	559	2028	6995	9184	-116.0318(3)	-115.9735(4)	1.586(14)	-116.3160(3)	-116.2566(3)	1.616(13)
	943	3288	8404	11396	-116.0432(4)	-115.9814(4)	1.681(14)	-116.3220(3)	-116.2598(3)	1.692(12)
	1384	5068	10592	14978	-116.0502(4)	-115.9890(4)	1.663(14)	-116.3231(3)	-116.2624(3)	1.653(10)
**CN19**	515	1886	8496	10615	-128.5631(4)	-128.5069(4)	1.529(16)	-128.8792(3)	-128.8239(3)	1.505(13)
	1487	4806	12876	15512	-128.5828(4)	-128.5242(4)	1.595(16)	-128.8872(3)	-128.8294(3)	1.572(13)

[a] Data from our work in Ref. 4.

In Table S7, we report the VMC and DMC total and vertical excitation energies obtained with CAS expansions. We employ the minimal CAS over π orbitals and, for the smaller CN3–CN5, also a larger space always over π orbitals. For CN11 and CN13, we truncate the wave functions using the same percentage of the total weight for the ground and excited states. For larger dyes, we do not compute the QMC-CAS excitation energies since the CASSCF computation becomes computationally too demanding.

Table S7: VMC and DMC total energies (a.u.) and excitation energies (eV) of cyanine dyes obtained for CAS wave functions. All the variational parameters (Jastrow, orbital, and CI coefficients) are optimized in energy minimization. The maug-cc-pVDZ basis set is employed.

System	CAS	No. det		No. parm		E_{VMC}		ΔE_{VMC}	E_{DMC}		ΔE_{DMC}
	(n,m)	GS	ES	GS	ES	GS	ES		GS	ES	
**CN3**	4,3	5	4	232	230	-28.3057(2)	-28.0238(3)	7.667(11)	-28.3693(2)	-28.0941(2)	7.488(8)
	4,6	113	112	302	293	-28.3077(4)	-28.0302(3)	7.552(13)	-28.3694(2)	-28.0959(2)	7.442(7)
**CN5**[a]	6,5	52	48	567	561	-40.8468(4)	-40.6583(4)	5.130(15)	-40.9433(3)	-40.7582(2)	5.038(10)
	6,10	7232	7168	3134	3064	-40.8498(4)	-40.6628(4)	5.090(15)	-40.9439(3)	-40.7594(3)	5.022(11)
**CN7**	8,7	625	600	1250	1220	-53.3750(4)	-53.2291(4)	3.969(15)	-53.5056(2)	-53.3649(3)	3.828(10)
	8,14	6802	5158	2675	2302	-53.3781(4)	-53.2421(4)	3.700(16)	-53.5057(2)	-53.3702(2)	3.686(6)
**CN9**	10,9	7956	7920	4257	4197	-65.9151(4)	-65.8007(4)	3.114(15)	-66.0728(2)	-65.9610(2)	3.042(9)
**CN11**[b]	12,11	7642	13360	3937	4914	-78.4485(4)	-78.3535(4)	2.585(14)	-78.6376(3)	-78.5436(3)	2.555(12)
**CN13**[c]	14,13	529	3338	3445	3949	-90.9798(4)	-90.9017(4)	2.130(14)	-91.1993(3)	-91.1204(3)	2.146(11)

[a] Data from our work in Ref. 4.
[b] Truncated so that, for each state, $\sum c_i^2 = 0.9985$ of the total wave function.
[c] Truncated so that, for each state, $\sum c_i^2 = 0.9765$ of the total wave function.

S5 Geometries

We include the geometries (Ang) of the longer cyanine chains, namely, CN13–CN19. We compute them at the PBE0/cc-pVQZ level as in Ref. 5. The geometries of the smaller molecules are taken from Ref. 5.

CN13

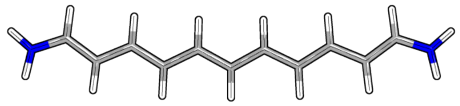

Figure S7: Schematic representation of CN13: 11 carbon (gray), 2 nitrogen (blue), and 15 hydrogen (white) atoms.

N	0.000000	-7.289060	-0.044934
N	0.000000	7.289060	-0.044934
C	0.000000	-6.023490	-0.447609
C	0.000000	6.023490	-0.447609
C	0.000000	0.000000	0.629127
C	0.000000	-1.212424	-0.040204
C	0.000000	1.212424	-0.040204
C	0.000000	-2.463906	0.567112
C	0.000000	2.463906	0.567112
C	0.000000	-3.632650	-0.163987
C	0.000000	3.632650	-0.163987
C	0.000000	-4.922661	0.369880
C	0.000000	4.922661	0.369880
H	0.000000	0.000000	1.714781
H	0.000000	-1.184395	-1.127980
H	0.000000	1.184395	-1.127980
H	0.000000	-2.519017	1.651245
H	0.000000	2.519017	1.651245
H	0.000000	-3.543691	-1.248548
H	0.000000	3.543691	-1.248548
H	0.000000	-5.054059	1.447094
H	0.000000	5.054059	1.447094
H	0.000000	-5.884505	-1.523814
H	0.000000	5.884505	-1.523814
H	0.000000	-8.044341	-0.704929
H	0.000000	8.044341	-0.704929
H	0.000000	-7.528784	0.931237
H	0.000000	7.528784	0.931237

CN15

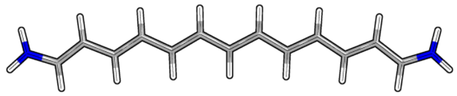

Figure S8: Schematic representation of CN15: 13 carbon (gray), 2 nitrogen (blue), 17 hydrogen (white) atoms.

N	0.000000	8.508297	0.217758
N	0.000000	-8.508297	0.217758
C	0.000000	7.233352	0.597940
C	0.000000	-7.233352	0.597940
C	0.000000	6.148232	-0.237196
C	0.000000	-6.148232	-0.237196
C	0.000000	0.000000	0.046299
C	0.000000	1.231663	-0.593462
C	0.000000	-1.231663	-0.593462
C	0.000000	2.426644	0.101443
C	0.000000	-2.426644	0.101443
C	0.000000	3.693293	-0.479589
C	0.000000	-3.693293	-0.479589
C	0.000000	4.846054	0.272596
C	0.000000	-4.846054	0.272596
H	0.000000	0.000000	1.134337
H	0.000000	1.256342	-1.679000
H	0.000000	-1.256342	-1.679000
H	0.000000	2.375489	1.188356
H	0.000000	-2.375489	1.188356
H	0.000000	3.769621	-1.562581
H	0.000000	-3.769621	-1.562581
H	0.000000	4.737161	1.355349
H	0.000000	-4.737161	1.355349
H	0.000000	6.298295	-1.312058
H	0.000000	-6.298295	-1.312058
H	0.000000	7.076055	1.671557
H	0.000000	-7.076055	1.671557
H	0.000000	8.765298	-0.753744
H	0.000000	-8.765298	-0.753744
H	0.000000	9.250963	0.891361
H	0.000000	-9.250963	0.891361

CN17

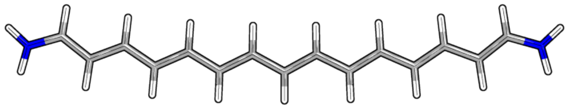

Figure S9: Schematic representation of CN17: 15 carbon (gray), 2 nitrogen (blue), and 19 hydrogen (white) atoms.

N	0.000000	9.725569	-0.125558
N	0.000000	-9.725569	-0.125558
C	0.000000	8.442671	-0.484888
C	0.000000	-8.442671	-0.484888
C	0.000000	7.371927	0.366212
C	0.000000	-7.371927	0.366212
C	0.000000	6.059321	-0.121564
C	0.000000	-6.059321	-0.121564
C	0.000000	0.000000	0.860798
C	0.000000	1.214675	0.194726
C	0.000000	-1.214675	0.194726
C	0.000000	2.462114	0.808691
C	0.000000	-2.462114	0.808691
C	0.000000	3.641589	0.091835
C	0.000000	-3.641589	0.091835
C	0.000000	4.921074	0.649315
C	0.000000	-4.921074	0.649315
H	0.000000	0.000000	1.946757
H	0.000000	1.191354	-0.893030
H	0.000000	-1.191354	-0.893030
H	0.000000	2.508181	1.893655
H	0.000000	-2.508181	1.893655
H	0.000000	3.570294	-0.993943
H	0.000000	-3.570294	-0.993943
H	0.000000	5.016284	1.730923
H	0.000000	-5.016284	1.730923
H	0.000000	5.932476	-1.202388
H	0.000000	-5.932476	-1.202388
H	0.000000	7.539033	1.438652
H	0.000000	-7.539033	1.438652
H	0.000000	8.268511	-1.555851
H	0.000000	-8.268511	-1.555851
H	0.000000	10.456414	-0.811463
H	0.000000	-10.456414	-0.811463
H	0.000000	9.998449	0.841307
H	0.000000	-9.998449	0.841307

CN19

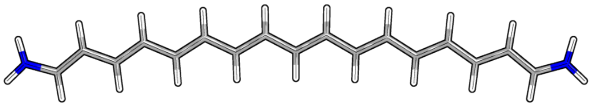

Figure S10: Schematic representation of CN19: 17 carbon (gray), 2 nitrogen (blue), 21 hydrogen (white) atoms.

N	0.000000	10.941184	0.524943
N	0.000000	-10.941184	0.524943
C	0.000000	9.651720	0.865931
C	0.000000	-9.651720	0.865931
C	0.000000	8.593827	0.000894
C	0.000000	-8.593827	0.000894
C	0.000000	7.272338	0.469053
C	0.000000	-7.272338	0.469053
C	0.000000	6.147153	-0.318167
C	0.000000	-6.147153	-0.318167
C	0.000000	0.000000	0.025660
C	0.000000	1.230886	-0.615165
C	0.000000	-1.230886	-0.615165
C	0.000000	2.430477	0.073420
C	0.000000	-2.430477	0.073420
C	0.000000	3.691209	-0.517584
C	0.000000	-3.691209	-0.517584
C	0.000000	4.856782	0.218345
C	0.000000	-4.856782	0.218345
H	0.000000	0.000000	1.113623
H	0.000000	1.252289	-1.701023
H	0.000000	-1.252289	-1.701023
H	0.000000	2.386918	1.160543
H	0.000000	-2.386918	1.160543
H	0.000000	3.756100	-1.601690
H	0.000000	-3.756100	-1.601690
H	0.000000	4.767841	1.302827
H	0.000000	-4.767841	1.302827
H	0.000000	6.259023	-1.398275
H	0.000000	-6.259023	-1.398275
H	0.000000	7.129678	1.547930
H	0.000000	-7.129678	1.547930
H	0.000000	8.775903	-1.069182
H	0.000000	-8.775903	-1.069182
H	0.000000	9.462705	1.934386
H	0.000000	-9.462705	1.934386
H	0.000000	11.227825	-0.437741
H	0.000000	-11.227825	-0.437741
H	0.000000	11.661787	1.221325
H	0.000000	-11.661787	1.221325

References

(1) Garniron, Y.; Applencourt, T.; Gasperich, K.; Benali, A.; Ferté, A.; Paquier, J.; Pradines, B.; Assaraf, R.; Reinhardt, P.; Toulouse, J.; Barbaresco, P.; Renon, N.; David, G.; Malrieu, J.-P.; Véril, M.; Caffarel, M.; Loos, P.-F.; Giner, E.; Scemama, A. Quantum Package 2.0: An Open-Source Determinant-Driven Suite of Programs. *Journal of Chemical Theory and Computation* **2019**, *15*, 3591–3609.

(2) Robinson, P. J.; Pineda Flores, S. D.; Neuscamman, E. Excitation variance matching with limited configuration interaction expansions in variational Monte Carlo. *The Journal of Chemical Physics* **2017**, *147*, 164114.

(3) Pineda Flores, S. D.; Neuscamman, E. Excited State Specific Multi-Slater Jastrow Wave Functions. *The Journal of Physical Chemistry A* **2019**, *123*, 1487–1497.

(4) Cuzzocrea, A.; Scemama, A.; Briels, W. J.; Moroni, S.; Filippi, C. Variational Principles in Quantum Monte Carlo: The Troubled Story of Variance Minimization. *Journal of Chemical Theory and Computation* **2020**, *16*, 4203–4212.

(5) Boulanger, P.; Jacquemin, D.; Duchemin, I.; Blase, X. Fast and Accurate Electronic Excitations in Cyanines with the Many-Body Bethe-Slapter Approach. *J. Chem. Theory Comput.* **2014**, *10*, 1212–1218.
